# The indirect pathway hypothesis of schizophrenia: insights from perinatal asphyxia

**DOI:** 10.3389/fphys.2026.1759386

**Published:** 2026-03-16

**Authors:** Pablo Vázquez-Borsetti

**Affiliations:** Institute of Cell Biology and Neurosciences (IBCN) – National Scientific and Technical Research Council (CONICET), Buenos Aires, Argentina

**Keywords:** indirect pathway, MSN, medium spiny neuron, perinatal asphyxia, schizophrenia spectrum disorders, thalamocortical dysregulation

## Abstract

The comorbidity between perinatal asphyxia (PA) and schizophrenia spectrum disorders (SSD) has been consistently documented in both clinical and experimental research. Individuals exposed to PA show an increased risk of developing long-term neuropsychiatric conditions, including SSD. Experimental models reveal that PA disrupts neurodevelopmental processes also altered in SSD. Moreover, converging evidence indicates that PA can affect the maturation and functional balance of basal ganglia circuits, particularly the indirect pathway, which relies heavily on D2 receptor–mediated signaling and is consistently implicated in the pathophysiology of SSD. Alterations in this pathway may therefore represent a mechanistic link contributing to the shared vulnerability between PA and SSD, where thalamic disinhibition resulting from indirect pathway dysfunction reduces the filtering of information relayed from the thalamus to the cortex, thereby facilitating the emergence of positive symptoms.

## Introduction

For some time now, our group’s working hypothesis has been that a deterioration of the indirect pathway of the basal ganglia may generate symptoms compatible with those observed in at least a subgroup of patients with schizophrenia spectrum disorders (SSD). This perspective is not unique to us; it also appears in the literature, sometimes implicitly and at other times more explicitly (Bernard et al., 2017; Jaaro-Peled et al., 2013; Macpherson et al., 2014; Niwa et al., 2013). Within the framework of our work, we have referred to this idea as the “indirect pathway hypothesis of schizophrenia.” This should not be confused with the use of the same term in psychiatry, where it mainly refers to mediated effects, for example, when a variable such as stress, childhood trauma influences psychiatric symptoms through an intermediate factor (an indirect effect).

In parallel, it is well established that perinatal asphyxia (PA) increases the risk of developing SSD (Laurens et al., 2015). Various studies have estimated this relative risk to be approximately fourfold (Dalman et al., 2001; Pugliese et al., 2019; Zornberg et al., 2000). Moreover, the degree of hypoxic damage correlates with the incidence of SSD (Cannon et al., 1999; 2000). PA not only increases vulnerability to SSD but also to other psychiatric disorders that often share overlapping symptoms with it (Giannopoulou et al., 2018). In addition, other conditions associated with PA, such as preterm birth, are also linked to a higher risk of schizophrenia (Nosarti et al., 2012). PA has also been associated with attention deficit hyperactivity disorder (ADHD) (Getahun et al., 2013) see also (Bentham et al., 2016), which, in turn, is highly overrepresented in SSD populations, with studies reporting ADHD in one-third to one-half of patients with SSD (Arican et al., 2019).

This link between PA and SSD raises the question of how exposure to perinatal hypoxia results in increased vulnerability to schizophrenia. In line with our hypothesis, human studies have shown that PA specifically affects subcortical structures, such as the basal ganglia and thalamus (Wortinger et al., 2022), and can produce motor symptoms consistent with damage in these regions (Kuiper et al., 2020). It has also been shown that PA can impact catecholaminergic nuclei in the midbrain (Pagida et al., 2013). PA is also associated with detectable cortical damage. This type of damage is very commonly reported in animal model studies (Vázquez-Borsetti et al., 2016; 2019), but it is less consistently observed in human studies, where it appears to be less severe than the damage to the basal ganglia (Heinz and Provenzale, 2009). In this context, structural MRI studies have identified similar morphological brain alterations in individuals exposed to PA and in patients with schizophrenia, when compared with controls (Wortinger et al., 2022). These alterations were particularly pronounced in the caudate nucleus.

## Schizophrenia as a spectrum and the complexity of its etiology

Most clinicians and researchers currently agree that the disorders grouped under the term *schizophrenia* are heterogeneous (Andreasen, 1982). Multiple hypotheses about the origin of schizophrenia have withstood the test of time. Among these are those focused on neurotransmitter alterations, such as dopamine excess or deficit, or changes related to serotonin (Carlsson and Carlsson, 2006). Other proposals point to the deterioration of specific brain structures, such as the prefrontal cortex or the hippocampus, which have given rise to different explanatory models, see (Luvsannyam et al., 2022). If we consider that this pathology may have multiple origins, this characteristic could explain the high variability observed in symptomatology. In this regard, alterations of the cortico–striato–thalamo–cortical loop, together with those of the catecholaminergic nuclei of the midbrain, encompass most of the existing hypotheses about SSD. This makes sense, since it is difficult to imagine that the functioning of such tightly interrelated structures could remain intact if deterioration occurs in any one link of the chain. Therefore, these hypotheses are not necessarily contradictory. In fact, they can be integrated into a common framework, in which the indirect pathway hypothesis does not compete with the others but rather functions as an integrative explanatory model that brings together several of the explanations proposed.

## The basal ganglia loop

**FIGURE 1:**
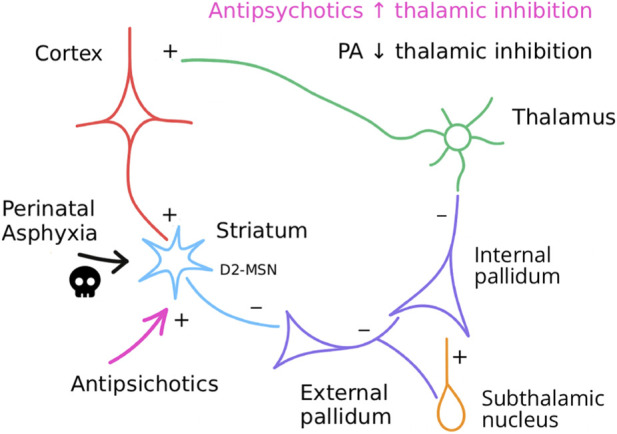
The indirect pathway of the cortico–striato–thalamo–cortical circuit, historically linked to movement disorders, also plays an essential role in cognitive processing. Impairment of a specific component, medium spiny neurons (MSNs) expressing dopamine D2 receptors, may underlie the increased vulnerability to schizophrenia spectrum disorders (SSD) observed after perinatal asphyxia (PA). While PA is proposed to diminish thalamic filtering, antipsychotic drugs produce the opposite effect by enhancing the filtering of information transmitted from the thalamus to the cortex.

The basal ganglia participate in the regulation of movement, cognition, and motivation through circuits that connect the cortex, striatum, globus pallidus, subthalamic nucleus, substantia nigra, and thalamus (Figure 1), forming the cortico–striato–thalamo–cortical loop. Functionally, two main pathways stand out: the direct and the indirect pathways.

The direct pathway facilitates the flow of information from the thalamus to the cortex. Cortical activation stimulates striatal medium spiny neurons (MSN) with D1 dopamine receptors, which inhibit the internal globus pallidus and the substantia nigra pars reticulata. Since these structures exert tonic inhibition on the thalamus, their suppression reduces this inhibition and allows the thalamus to enhance cortical activity (Van Beest et al., 2024).

In contrast, the indirect pathway contributes to the suppression of information flow from the thalamus to the cortex. In this pathway, the cortex activates a different subgroup of MSNs typically expressing D2 dopamine receptors, which inhibit the external globus pallidus. This structure then disinhibits the subthalamic nucleus, which in turn excites the internal globus pallidus and the substantia nigra pars reticulata, thereby increasing inhibition of the thalamus and reducing cortical excitation, see (Bertran-Gonzalez, 2010; Nishi and Snyder, 2010).

Dopamine plays a modulatory role that always favors the flow of information from the thalamus to the cortex: it stimulates the direct pathway via excitatory D1 receptors and attenuates the indirect pathway via inhibitory D2 receptors, thus jointly promoting the initiation and maintenance of behavior. An appropriate balance between both pathways is necessary not only for motor control but also for cognitive and emotional functions (Obeso et al., 2014). Within this framework, it seems plausible that alterations of the indirect pathway could contribute to the pathophysiology of SSD, particularly to deficits in information filtering and the emergence of positive symptoms. It is reasonable to think that an overabundance of information in the thalamocortical pathway may generate disorganization, hallucinations, and/or delusions.

## SSD and basal ganglia dysfunction

Several studies have examined the relationship between basal ganglia dysfunction and SSD, see (Deserno et al., 2016; McCutcheon et al., 2019). Among them, the comprehensive review by Perez-Costas et al. (2010) provides an extensive compilation of anatomical and neurochemical alterations in the basal ganglia associated with dopaminergic dysregulation in schizophrenia. The authors emphasize that dopamine exerts differential effects depending on receptor subtype and extrasynaptic mechanisms, and that it plays a central role in functions typically impaired in SSD, including psychosis, cognitive decline, and reward processing. Moreover, the review highlights evidence suggesting that dopaminergic dysregulation within the basal ganglia is intrinsic to the disorder, may precede the onset of psychosis, and constitutes a risk factor for illness development. The work calls for further research linking these alterations to clinical symptoms, genetic liability, and their possible continuity with related neuropsychiatric conditions such as bipolar disorder and major depression. However, the review does not aim to provide a detailed mechanistic account of how or why these dopaminergic alterations translate into the specific behavioral and cognitive symptoms observed in patients.

The meta-analysis by Bernard et al. (2017) and colleagues demonstrated robust functional alterations of the basal ganglia in schizophrenia. Using an activation-likelihood-estimation approach across 42 functional neuroimaging studies spanning multiple task domains, this work showed that patients with schizophrenia exhibit markedly reduced basal ganglia activation relative to healthy controls, indicating a broad and domain-general dysfunction of these circuits. However, while these findings strongly implicate basal ganglia involvement in the disorder, they do not explicitly attribute this dysfunction to specific pathways within the basal ganglia, such as the indirect pathway.

A recent study (Tang et al., 2024) examined the relationship between the globus pallidus and schizophrenia spectrum disorders, reporting an increase in the volume of this structure in patients with SSD. The authors identify the indirect pathway as a key circuit involved in the regulation of human behavior; however, they do not elaborate in detail on the potential mechanisms through which alterations in this pathway might contribute to the generation of clinical symptoms.

Another recent study has associated catatonia, a symptom frequently observed in patients with schizophrenia, with the indirect pathway (Seif, 2025). The notion that the indirect pathway plays a central role appears to be implicitly present throughout much of the literature, and making this premise explicit would help to clarify both its strengths and its limitations.

## Effects of antipsychotic treatments

Antipsychotic drugs exert their main action by blocking D2 dopaminergic receptors (Spangemacher et al., 2025). This has direct consequences for basal ganglia circuitry, particularly the indirect pathway, as D2 receptors constitute one of the most abundant dopamine receptor populations in the brain (Gurevich, 1999; Suhara et al., 1999).

The blockade of D2 receptors by typical antipsychotics, such as haloperidol, disinhibits the indirect pathway and strengthens thalamic inhibition. This mechanism may contribute to the reduction of positive symptoms in schizophrenia by restoring information filtering and decreasing cortical hyperexcitability. This same blockade of dopaminergic receptors also contributes to the emergence of adverse side effects. Subsequently, a new generation of antipsychotics, referred to as atypical antipsychotics, was developed, allowing a similar therapeutic effect to be maintained with reduced dopaminergic receptor blockade, in part due to their broader pharmacological profile, including affinity for serotonergic receptors.

The mechanism of action of atypical antipsychotics remains incompletely understood, although it has been proposed to involve modulation of cortical 5-HT2A receptors, which are connected to circuits projecting to mesencephalic catecholaminergic nuclei (Vazquez-Borsetti et al., 2009), including both the ventral tegmental area and the dorsal raphe nucleus. In fact, it has been reported that some cortical neurons bifurcate their axons, providing innervation to both the ventral tegmental area and the dorsal raphe (Vázquez-Borsetti et al., 2011). Overall, the pharmacological binding profile of atypical antipsychotics allows them to achieve a therapeutic effect comparable to that of typical antipsychotics but with lower D2 receptor occupancy. D2 receptors still play a fundamental role in the therapeutic effect, yet lower occupancy helps mitigate parkinsonian-like side effects. It is worth mentioning the new generation of atypical antipsychotics, such as lurasidone, which retain affinity for D2 and 5-HT2A receptors but also display high affinity for the 5-HT7 receptor. The latter is associated with a specific subpopulation of D2-expressing medium spiny neurons (D2-MSNs), suggesting a more selective modulatory profile within the striatal circuitry (Fukuyama et al., 2022).

## Motor alterations in SSD and PA

The most significant side effect of antipsychotic drugs results from the blockade of D2 dopaminergic receptors in the striatum, which reduces dopaminergic activity in the nigrostriatal pathway and produces motor symptoms similar to those observed in Parkinson’s disease (antipsychotic-induced parkinsonism). Moreover, chronic compensatory overactivation of D2-MSN neurons may lead to toxicity and long-term neural alterations, giving rise to a characteristic motor disorder known as *tardive dyskinesia,* see (D’Abreu and Friedman, 2018).

The fact that antipsychotic drugs themselves can induce motor disturbances has somewhat masked the observation that motor symptoms also occur in comorbidity with SSD. This has been supported by evidence from untreated patients, see (Moura et al., 2024). Other research has demonstrated that these alterations constitute an intrinsic component of the neurodevelopmental disorder in SSD, besides than being side effects of D2 receptor–blocking agents (Cannon et al., 2002).

A high incidence of movement disorders has also been observed among relatives of patients, suggesting a genetic component affecting the motor system, possibly mediated by alterations in the nigrostriatal pathway (Koning et al., 2010). Thus, a family history of schizophrenia predicts poorer performance on motor tasks; likewise, low motor performance also serves as a predictor of the onset of psychotic symptoms (Burton et al., 2023). It is common for patients with schizophrenia to exhibit motor symptoms resembling parkinsonism or dyskinesias. Neuroimaging studies have shown that these two symptom profiles appear to have distinct characteristics that separate patients into two groups: dyskinesias are associated with alterations in the putamen and cerebellum, whereas parkinsonism is linked to more pronounced changes in the caudate and cerebral cortex (Sakreida et al., 2022). The other side of the coin is that the incidence of psychosis in Parkinson’s disease is remarkably high, reaching 26% according to a study using conservative criteria (Mack et al., 2012). Although this might initially seem inconsistent with the indirect pathway hypothesis, since reduced dopamine should intensify inhibitory filtering within this pathway, it is noteworthy that psychotic symptoms have been significantly associated with disease progression. This association is particularly meaningful considering the reported degeneration of striatal MSNs in late Parkinson’s disease (Zaja-Milatovic et al., 2005). Regarding the relationship between PA and Parkinson’s disease, we have not found any clinical studies directly linking them. However, postmortem studies in neonates have demonstrated that PA affects dopaminergic neurons in the midbrain (Pagida et al., 2013). This finding has implications also for SSD, as it suggests an alternative mechanism through which PA may disrupt the thalamo–meso–cortico–limbic loop. In parallel, the hypoxia-inducible factor 1 alpha (HIF-1α) pathway has been implicated in several pathogenic mechanisms underlying Parkinson’s disease and has consequently been proposed as a promising therapeutic target (Lestón Pinilla et al., 2021).

Another pathology linking PA, basal ganglia dysfunction, and SSD is Huntington’s disease, a hereditary neurodegenerative disorder that primarily affects striatal neurons. This condition illustrates the functional consequences of selective vulnerability of indirect-pathway MSNs. Early degeneration of D2-MSNs reduces inhibitory control over thalamocortical activity, resulting in motor disinhibition and choreiform movements, whereas later involvement of direct-pathway neurons contributes to the progression toward hypokinetic symptoms. This disease shows comorbidity with psychotic symptoms in 17.6% of cases (Connors et al., 2020). Also, antipsychotic drugs are a key therapeutic option for the treatment of chorea, as they ameliorate choreic movements among other symptoms (Coppen and Roos, 2017). Although Huntington’s chorea has a clear genetic basis, neurodevelopmental insults such as PA have been reported to precipitate an earlier onset of clinical symptoms (Barkhuizen, 2017). Another characteristic feature of the disease is the presence of evident damage in calbindin-expressing neurons within the basal ganglia (Seto-Ohshima et al., 1988). PA is a smoking gun pointing to environmental factors involved in motor alterations and SSD. In this sense, it is not unreasonable to propose that these processes involve a shared underlying mechanism.

Although ADHD is not strictly a motor disorder, it is closely linked to motor system dysfunction. Its clinical presentation includes motor hyperactivity, bodily restlessness, difficulties in inhibiting movements, impaired regulation of motor timing, and mild motor clumsiness, which is nonetheless observed in a substantial proportion of patients (Shimko and James, 2025). As mentioned above, ADHD also exhibits a high comorbidity with SSD and PA and has been consistently associated with reduced volumes in the frontal lobes, the striatum, and the white matter tracts connecting these regions (Cupertino et al., 2020). It is worth mentioning that the “indirect pathway hypothesis” for ADHD is explicitly and directly stated in the literature, see (Fu et al., 2025).

## Common genetic vulnerability for PA and SSD

Multiple genes implicated in the pathophysiology of PA also contribute to the etiology of SSD. Notable examples include *BDNF* (Brain-Derived Neurotrophic Factor), *MIF* (Macrophage Migration Inhibitory Factor), *DISC1* (Disrupted in Schizophrenia 1), *RELN* (Reelin), and the chromosomal region 10q24.32–33, which contains the *CNNM2* gene (Gören, 2016; Li et al., 2023; Okazaki et al., 2018; Scheepens et al., 2003).

- *BDNF* plays an important role in neuroprotection during PA (Han and Holtzman, 2000) and is essential for neuronal survival, differentiation, and synaptic plasticity. Altered *BDNF* expression has been linked to structural and functional impairments in the cortex and ventral tegmental area in patients with schizophrenia (Chandra et al., 2025). Although *BDNF* is not produced in large quantities within the striatum, it is transported via afferent axons and is vital for the survival of MSNs (Baydyuk and Xu, 2014). This suggests that dysfunction in connected brain regions may indirectly impair striatal function through disrupted *BDNF* signaling.
- *MIF* is a hypoxia-responsive gene. A promoter polymorphism in *MIF* has been associated with bronchopulmonary dysplasia, a common complication of prematurity, and has also been linked to SSD (Prencipe et al., 2011). This relationship may involve interactions with the hypoxia-inducible factor (HIF) pathway, highlighting a potential mechanism by which hypoxia-related genetic vulnerabilities influence neurodevelopmental outcomes (Okazaki et al., 2022).
- *DISC1* is a well-established genetic risk factor for schizophrenia. Initially identified in a Scottish family (Blackwood et al., 2001) with high rates of psychiatric disorders, it has since been linked also to PA. The stability of the DISC1 protein is affected by hypoxic conditions, suggesting that perinatal insults may exacerbate its dysfunction, thereby increasing susceptibility to SSD (Kawikova et al., 2024).
- *RELN*, which encodes a protein critical for neuronal migration, cortical development, and GABAergic signaling, has also been associated with both PA and SSD (Ovadia and Shifman, 2011). Specific haplotypes of *RELN* are linked to structural alterations in the striatum and increased vulnerability to schizophrenia (Pardo et al., 2023). Animal models have demonstrated that PA can induce pathological changes in Reelin-expressing neurons of the prefrontal cortex, further supporting its role in both conditions (Vázquez-Borsetti et al., 2016).
- The *CNNM2* gene, located in the 10q24.32–33 region, encodes a magnesium transporter involved in neuronal excitability and synaptic function. It has been consistently identified as a strong genetic risk factor for schizophrenia (Zhou et al., 2024), and its expression is influenced by PA, linking the contribution of this locus to both conditions (Paparelli et al., 2017).

A common pattern emerges among these genetic factors: they are frequently involved in neuroprotection, neuronal migration, and neurodevelopment. Several also implicate the mesolimbic pathway, suggesting that shared mechanisms related to hypoxia response, synaptic integrity, and circuit formation may underlie the comorbidity between PA and schizophrenia.

## Sex differences in vulnerability to asphyxia and schizophrenia

A consistently observed factor is the greater vulnerability to asphyxia in males. This has been documented in humans (Yu et al., 2022) and is also replicated in animal models, see (Murden et al., 2019), with some studies indicating that male newborns are disproportionately affected by perinatal complications, including asphyxia.

In the case of SSD, a similar pattern can be observed. The incidence of schizophrenia is higher in men than in women, with an approximate ratio of 1.4 to 1 (Li et al., 2022). The peak incidence in men occurs around the age of 22. In women, this peak is less pronounced; they show a flatter age pattern with a higher number of cases emerging after the age of 40, at which point the incidence surpasses that in men (Sommer et al., 2020).This phenomenon has been largely attributed to the protective effects of estrogen in women, see (Brand et al., 2021). The decline in estrogen levels in women during peri-menopause and menopause is thought to remove this protective factor, contributing to the later onset and the shift in incidence rates. Consistent with this view, estradiol has been shown to exert robust neuroprotective effects against hypoxia–ischemia in the neonatal brain across multiple structures, including the basal ganglia (Nuñez et al., 2007; Zhu et al., 2012). A protective effect of estradiol has also been reported in Huntington’s disease (Túnez et al., 2006). As discussed above, MSNs constitute the core pathological substrate of Huntington’s chorea. Notably, a growing body of literature describes sexual dimorphism in the striatum that specifically affects MSNs, a phenomenon that has also been implicated in sex-dependent behavioral differences (Krentzel and Meitzen, 2018). Taken together, these findings make it biologically plausible that estradiol may exert a shared protective mechanism in both SSD and PA by preserving MSNs, including those participating in the indirect pathway.

## Insight from animal models about the relationship between PA, SSD and the indirect pathway

The use of animal models to study SSD has been controversial as many of the disorder’s core symptoms are difficult to assess in non-human species. Despite these challenges, murine models of PA have been proposed as valid models of SSD. Measurements targeting negative-like symptoms, such as social isolation, have yielded consistent results (Laviola et al., 2004; Vázquez-Borsetti et al., 2016).

Prepulse inhibition, a phenomenon disrupted in SSD, refers to the ability of a weak prepulse to reduce the startle response elicited by a subsequent, more intense stimulus. This deficit is typically associated with positive symptoms, and in animal models it is widely used for antipsychotic drug screening. This phenomenon is also impaired in rats exposed to PA (Laplante et al., 2012). Notably, the study also reported increased male vulnerability, a feature common to both PA and SSD and previously discussed in this manuscript.

Beyond the value of PA as a model of SSD, the literature shows numerous similarities and parallels between the two conditions. A growing body of evidence supports a mechanistic link between PA, striatal developmental disturbances, and behavioral phenotypes associated with SSD. Experimental studies in rodents have shown that PA induces marked reductions (≈48%) in MSNs (Galvin and Oorschot, 2003). Using an independent model of PA, these findings were subsequently confirmed, and we further observed that calbindin-positive neurons are selectively affected in both the dorsal and ventral striatum (Vázquez-Borsetti et al., 2020). Colocalization of D2 receptors and calbindin has been reported in the striatum (Gangarossa et al., 2013), supporting the participation of calbindin-expressing neurons in the indirect pathway and their association with the D2-MSN population (Davis and Puhl, 2011). Other markers of D2-MSNs include the expression of *Htr7*, which encodes the serotonin receptor 7, and *Cartpt*, a cocaine- and amphetamine-regulated transcript (Gokce et al., 2016). Notably, *Htr7* harbors haplotypes that have been associated with schizophrenia (Ikeda et al., 2006).

Dysfunction of D2-MSNs in the indirect pathway reduces inhibitory output to the external globus pallidus. This increases inhibition of the internal globus pallidus, which in turn disinhibits the thalamus. The resulting overactivation of thalamocortical circuits may contribute to the motor and cognitive dysregulation observed in SSD and related disorders. The loss of calbindin-positive neurons is also accompanied by an increased density of calretinin-positive neurons, suggesting the activation of compensatory neurodevelopmental programs in response to early-life injury (Vázquez-Borsetti et al., 2020).

Comparable alterations have been reported in the anterior insular cortex, where PA induces a reduction of calbindin-positive neurons in layer II together with an atypical increase in Reelin-positive interneurons (Vázquez-Borsetti et al., 2019). This profile reinforces the idea that early hypoxic-ischemic insults elicit plastic, yet potentially maladaptive, responses from neuronal populations that are less directly affected at the time of the injury.

The impact of hypoxic–ischemic injury depends strongly on the timing of the insult relative to brain development. Injury may occur during gestation, in association with preterm or term birth, or in the early postnatal period. At each of these time points, the brain undergoes ongoing maturation processes, including neuronal migration, synaptogenesis, and circuit formation, which shape patterns of neural vulnerability and determine the susceptibility of specific brain regions and cell populations. GABAergic neurons originating from the ganglionic eminences reach the cortex and striatum in successive waves, a process that spans both gestational and early postnatal periods. In the cortex, these inhibitory neurons integrate into local circuits as interneurons (Guo and Anton, 2014; Lim et al., 2018). In the striatum, they give rise to both interneurons and projection neurons such as MSNs (Lebouc et al., 2020). These neuronal populations can be further subdivided into distinct subpopulations with specific developmental trajectories, which may therefore be differentially affected depending on the timing of the hypoxic insult. Early-migrating subpopulations, including neurons expressing parvalbumin and somatostatin (the latter also encompassing calbindin-positive neurons), are more likely to be compromised when injury occurs during their migratory or differentiation window. In contrast, later-developing populations, such as calretinin-positive and Reelin-positive interneurons, may be less affected or may undergo compensatory expansion. This may result in alterations in local circuit modulation by interneurons in both the cortex and the striatum. In addition, projection neuron populations such as MSNs may also be affected, potentially altering the balance between interneurons and projection neurons in the striatum.

These temporal dynamics introduce significant complexity when interpreting animal models, particularly given the well-recognized difficulties in establishing precise correspondences between stages of brain development in rodents and humans (Campos Eusebi et al., 2024; Ortiz et al., 2022; Williams and Riedemann, 2021). This temporal vulnerability also contributes to the heterogeneity of long-term neurological and psychiatric outcomes of PA preterm infants. Neuroimaging studies in humans consistently identify the hippocampus, basal ganglia, thalamus, and brainstem as the key regions for assessing the severity of PA-related gray matter damage, with both the pattern and the degree of injury depending strongly on the level of brain maturation at the time of the insult (Logitharajah et al., 2009; Mohammad et al., 2025).

Other example of the long-term functional consequences of PA comes from studies showing that affected rats display methamphetamine-induced hyperlocomotion in adulthood (Wakuda et al., 2008). This behavioral phenotype, which resembles the dopaminergic sensitization observed in schizophrenia, represents a delayed manifestation of the early insult. Notably, the exaggerated locomotor response is accompanied by increased dopamine release in the nucleus accumbens, indicating a persistent dysregulation of the mesolimbic dopaminergic pathway.

The newborn rat is often considered developmentally comparable to the preterm human infant; however, this assumption may not fully capture the complexity of human neurodevelopment. Nevertheless, this view is not incompatible with the indirect pathway hypothesis discussed above, given that preterm birth is associated with an increased risk of developing schizophrenia. In order to model a broader group of newborns affected by PA, alternative animal approaches have been proposed, including murine models in which the timing of the insult is delayed until GABAergic development more closely approximates that of humans. However, this strategy entails important limitations, as it overlooks critical biological processes associated with the transition to extrauterine life, which are tightly linked to PA pathophysiology (Ortiz et al., 2022).

Nevertheless, GABAergic developmental windows are relatively broad in both humans (Xu et al., 2011) and rodents (Topchiy et al., 2024), and both term and delayed modeling strategies have been shown to induce alterations in GABAergic neurons (Nisimov et al., 2018; Wang et al., 2011), including striatal MSNs (Ji et al., 2023).

Consistent with these observations, evidence from human studies in patients with a history of PA also supports the presence of alterations in GABAergic neurons (Robinson et al., 2006). In larger animal models, abnormalities in MSNs have likewise been reported in the caudate nucleus of preterm sheep following *in utero* global cerebral hypoxia–ischemia (McClendon et al., 2014).

Taken together, these findings highlight the complexity involved in translating results from animal models to the human condition. At the same time, they underscore the substantial methodological constraints that limit the acquisition of direct evidence in humans supporting selective damage of the indirect pathway following PA and its potential relationship with schizophrenia spectrum disorders. This limitation should therefore be taken into account when interpreting both experimental and clinical findings. Nevertheless, converging findings from multiple experimental and clinical lines of research increasingly support the involvement of the indirect pathway in SSD and its relationship with PA, thereby reinforcing the relevance of this framework despite the aforementioned methodological limitations.

## Final discussion

Taken together, the evidence presented here provides a framework for understanding how PA disrupts the maturation of inhibitory circuits within the striatum and the broader corticolimbic and corticothalamic loops, highlighting the selective vulnerability of MSNs. These developmental disturbances converge on key nodes of the basal ganglia circuitry, particularly the indirect pathway, whose dysfunction can lead to thalamic disinhibition and thereby contribute to cognitive disorganization and positive symptoms. This hypothesis is not proposed as the sole mechanism through which SSD may develop and it does not contradict other etiological models of the disorder; rather, it is compatible with them and may represent one component within a multifactorial neurodevelopmental framework. It is also important to note that the high degree of interconnection among these structures makes it unlikely that damage to one node would occur without downstream effects on others.

Another potentially useful implication of this framework is the identification of new therapeutic targets and/or alternatives capable of reducing side effects. Such targets could include drugs acting at the level of the globus pallidus or the subthalamic nucleus, which may be beneficial for patients who show resistance to antipsychotics. With regard to side effects, particularly motor symptoms, it would be interesting to search for differential gene expression between the motor and cognitive pathways as a means to develop more selective treatments with fewer motor side effects.

From this perspective, PA and antipsychotic drugs exert opposite effects on the indirect pathway of the basal ganglia: the former tends to impair it, whereas the latter enhance it. Antipsychotics block dopamine-mediated inhibition of indirect-pathway MSNs, thereby increasing their activity and allowing downstream circuitry to inhibit the thalamus. In contrast, perinatal asphyxia leads to the loss of these neurons, preventing them from fulfilling their regulatory function. Continued progress in the prevention and treatment of PA, such as the use of therapeutic hypothermia, together with advances in interventions for other developmental insults, may help reduce the proportion of individuals who go on to develop long-term neuropsychiatric sequelae, including SSD.
